# Regulatory role of exogenous 24-epibrassinolide on tomato fruit quality

**DOI:** 10.1186/s12870-025-06710-8

**Published:** 2025-05-27

**Authors:** Shilei Luo, Xianxia He, Long Li, Zeci Liu, Guobin Zhang, Jian Lv, Jihua Yu

**Affiliations:** 1 College of Horticulture, Gansu Agricultural University, Lanzhou, Gansu 730070 China; 2 State Key Laboratory of Aridland Crop Science, Lanzhou, Gansu 730070 China

**Keywords:** Brassinolide, Fruit quality, Sugar and acid metabolism, Volatile substances

## Abstract

**Background:**

Brassinosteroids (BRs) are known to regulate fruit development, ripening, and metabolic processes in plants. In this study, the impact of exogenous 24-epibrassinolide (EBR) on tomato fruit quality was examined using ‘Micro-Tom’ tomatoes.

**Results:**

Treatments included control (CK), EBR, and brassinazole (Brz, BR biosynthesis inhibitor). EBR application accelerated fruit ripening, evidenced by decreased fruit hardness and increased soluble solids and vitamin C (VC) content. EBR enhanced glucose and fructose accumulation and upregulated key genes involved in sugar metabolism (*SS*, *NI*, *SPS*, *AI*). Conversely, Brz treatment inhibited these effects. EBR also reduced malic and citric acid levels by downregulating genes associated with acid metabolism (*CS*, *PPC1*, *PPC*2, *MDH*), while Brz increased acid content. Furthermore, EBR significantly elevated flavonoid compounds, such as rutin and quercetin, and altered volatile profiles as detected by electronic nose analysis, particularly affecting W2W and W5S sensors.

**Conclusion:**

These results indicated that EBR could effectively modulate sugar and acid metabolism, enhance flavonoid content, and influence fruit aroma, suggesting its potential to improve tomato fruit quality.

## Introduction

Tomatoes(*Solanum lycopersicum* L.), a significant vegetable in human diets, offer substantial nutritional and economic value [1]. The high levels of antioxidants in tomatoes, such as vitamin C and lycopene, play a crucial role in human health, including enhancing immunity and preventing cancer [2, 3]. The quality of tomato fruits is determined by both nutritional and appearance qualities, which are key factors influencing consumer acceptance and recognition [4]. Enhancing the quality of tomatoes not only increases consumer choice but also improves economic returns and boosts income [5]. The quality of tomato fruits is influenced by genetic factors, environmental conditions, and cultivation techniques. Appropriate cultivation techniques is crucial for enhancing the yield and quality of tomatoes. Balanced fertilization, foliar fertilizers, organic amendments, and biochar application collectively enhanced tomato yield and quality while improving soil health [6–9]. Appropriate water deficit could increase the content of soluble solids, sugars, acids, vitamin C, and amino acids in tomatoes, and enhance their characteristic aroma [10, 11]. Similarly, moderate salt stress could also enhance the nutritional value of tomatoes, such as soluble solids, proteins, and sugars, and significantly increase the content of volatile compounds (alcohols, aldehydes, esters, etc.) [12, 13]. Moreover, Supplemental lighting during morning and evening hours enhances the accumulation of vitamin C, organic acids, amino acids, carotenoids, phenolic acids, flavonoids, and aroma compounds in tomato fruits, thereby improving fruit quality [14]. These findings highlight the importance of integrated strategies in ensuring optimal growth conditions and superior fruit quality in tomatoes. Currently, the pursuit of higher yields has often come at the expense of the original flavor [15–17]. Therefore, quality enhancement is a key direction for future tomato cultivation and breeding efforts.

Tomatoes are rich in nutrients, containing a variety of mineral elements, vitamins, polyphenols, organic acids, soluble sugars, and carotenoids, including lycopene and β-carotene [17]. The sugar content significantly influences tomato quality and consumer preference [18], with fructose and glucose being the predominant flavor sugars present [19]. The sourness of tomatoes is primarily attributed to citric acid and malic acid, which together account for 90% of the total organic acids [20]. Hexokinase (HXK), sucrose synthase (SS), alkaline/neutral invertase (NI), sucrose phosphate synthase (SPS), acid invertase (AI), pyruvate kinase (PK), fructose-1, 6-bisphosphate (FBP), phosphoenolpyruvate carboxykinase (PEPCK) are all key enzymes in fruit sugar accumulation and metabolism [18, 21–23]. Similarly, citrate synthase (CS), phosphoenolpyruvate carboxylase (PPC), and malate dehydrogenase (MDH) are main enzymes in the acid metabolism pathway, playing an important role in the synthesis and accumulation of fruit acid components [24]. Flavonoids, as a class of secondary metabolites, play essential roles within biological systems, with tomato flavonoids mainly including naringenin chalcone, rutin, and kaempferol [25]. The aroma of tomatoes originates from volatile aromatic compounds; over 400 distinct volatile compounds have been identified to date, predominantly consisting of alcohols, aldehydes, ketones, terpenes, esters, and sulfur-containing compounds [17].

Brassinosteroids (BRs) are vital plant steroid hormones that play a key role in coordinating fundamental growth and developmental processes in plants [26]. Research indicate that their physiological mechanisms were primarily manifested in regulating seed germination, flowering time, senescence, tropic responses, photosynthesis, and stress resistance [27]. BRs also promoted fruit ripening and enhanced crop yield and quality, earning them the designation of “multifunctional hormones“ [28–30]. In the context of grape quality regulation, BRs could improve both external quality (increasing fruit diameter, modulating firmness, augmenting individual fruit weight, and enhancing fruit color) and internal quality (optimizing sugar, organic acid, aromatic compounds, anthocyanin, and antioxidant levels) [31, 32]. Furthermore, during tomato fruit development, the application of BRs effectively induced fruit ripening, elevating soluble sugar, ascorbic acid, and lycopene content, while also enhancing respiration rates and ethylene production [33]. In strawberries, exogenous application of BRs increased individual fruit weight and improved sugar content, soluble solids, ascorbic acid, and anthocyanin levels, with particularly pronounced affected under adverse conditions in subtropical regions [34].BR also could enhance the transcription levels of genes involved in the biosynthesis of theanine, thereby alleviating the negative impact of moderate heat stress on tea quality [35]. Notably, as a synthetic BR analog, 24-epibrassinolide (EBR) exhibits greater stability and broader biological activity, demonstrating superior regulatory effects compared to BR in various crops, while maintaining low toxicity and high efficiency [36–41]. In summary, as a multifunctional plant hormone, BR regulate crop growth and metabolic processes through various pathways, holding significant application value in improving crop yield and quality [42].

Numerous studies have demonstrated that BRs play a crucial role in regulating crop stress resistance and enhancing yield and quality. In this experiment, the application of EBR during the green mature stage of tomato fruit was found to promote fruit coloration. Analysis of quality indicators such as soluble solids, vitamin C, sugar, and acid components revealed that EBR can facilitate tomato maturation and improve fruit quality, providing valuable insights for future high-quality tomato cultivation.

## Materials and methods

In this experiment, Micro-Tom’ Tomato (*Solanum lycopersicum* L.) was used as the experimental material. The seeds were placed in triangular flasks and 50 mL of distilled water was added. The flasks were placed on a shaker at 28℃ and 165 rpm until germination. After germination, the seeds were transferred to cultivation pots containing substrate and regularly watered with 1/2 Hoagland nutrient solution. From the fruiting period onwards, the plants were watered with 1 times Hoagland nutrient solution until the fruit was mature.

A total of three treatments were established for the experiment: CK (distilled water + 0.01% Tween-80), EBR (0.15 mg/L EBR + 0.01% Tween-80), and Brz (4 µmol/L Brz + 0.01% Tween-80). Each treatment included three replicates, with ten tomato plants per replicate. During the tomato fruit expansion period, plants with consistent growth, pollination time, and uniform fruit size and color without disease or insect damage were selected for treatment. The treatment involved coating the fruit surface with EBR, with the criterion being that all fruit surfaces were moistened with condensed water droplets without dripping. The coating was repeated every three days, and samples were taken on the 5, 10, 15, 20, and 25th d after the first treatment, followed by measurements of fruit quality and other related indicators.

### Determination of tomato fruit hardness

The GY-4 digital fruit hardness tester was used to determine the fruit hardness, with 3 replicates for each treatment.

### Determination of soluble solids and vitamin C content

Use PAL-1 handheld refractometer (ATAGOCO., LTD, Japan) to determine soluble solids in tomato fruits, with 3 replicates for each treatment [43].

The VC content was determined using the 2, 6-dichlorophenol indophenol sodium staining method. We weighed 1.0 g of fresh tomato pulp sample, added a small amount of quartz sand and 3 mL of 2% oxalic acid, washed with 1% oxalic acid, filtered into a 100 mL volumetric flask, then added 1 mL of 30% zinc sulfate and 1 mL of 15% potassium ferrocyanide, supplemented with 1% oxalic acid, shaken well, and filtered. Finally, we added 2 mL of dye and 5 mL of xylene to 4 mL of filtrate and measured the OD value of the mixture at 500 nm [43].

### Determination of sugar and acid components in tomato fruits

The determination of sugar and acid components follows the method of Li et al. [24]. The extraction and determination of the content of sugar components is based on the method previously optimized in our laboratory. A precisely weighed 5 g fresh sample was ground into homogenate, which was then transferred to a 50 mL centrifuge tube and diluted with ultrapure water to 25 mL. The slurry was subjected to ultrasonic extraction for 60 min at a temperature of 30℃, followed by centrifugation at 4℃ and 10, 000 rpm for 10 min. Two milliliters of the supernatant were filtered through a 0.22 μm microporous membrane, and the filtrate was analyzed using high-performance liquid chromatography (HPLC). The detector used was an Agilent series 1100 diode array detector (DAD), and the chromatographic column was an LC-NH2 (460 mm × 250 mm) column. The column temperature was maintained at 30℃, and the mobile phase was composed of acetonitrile and water in a ratio of 75: 25. The mobile phase was degassed using ultrasound for 15 min, and the flow rate was set at 1.0 mL/min with an injection volume of 20 µL. Each sample was analyzed in triplicate, and the mean value was calculated.

Weigh 5.0 g of fresh and clean tomato sample and transfer it to a 50 mL centrifuge tube. Dilute it to 25 mL with ultrapure water and homogenize it by centrifugation for 10 min. Then, take 1 mL of the supernatant and filter it into a 2 mL brown sample bottle through a disposable aqueous membrane with 0.22 μm. The filtrate is used for HPLC with a Hi PiexH (300 mm × 7.7 mm) column and a UV detector. The mobile phase is sodium dihydrogen phosphate, with a concentration of 0.2 mmol·L^− 1^, a flow rate of 0.5 mL·min^− 1^, a column temperature of 30 °C, and an injection volume of 10 µL.

### Determination of flavonoid content in tomato fruit

The extraction and determination of phenolic flavonoids follow the method of Jin et al. [44]. Firstly, take a freeze-dried sample of tomato fruit (0.1 g) and extract it evenly in 1 mL of methanol for 2 h. Shake continuously during the extraction process to ensure complete extraction. Centrifuge the extraction solution at 8000 rpm and continue for 10 min. Remove the supernatant and pass it through a disposable filter membrane into a brown bottle for measurement. The HPLC conditions were a C18 column (250 mm × 4.6 mm, 5 μm, Agilent, Milford, Massachusetts, USA) with a column temperature of 30 °C and a sample volume of 10 µL. The mobile phase A was methanol, and the mobile phase B was 1% (v/v) acetic acid, with a flow rate of 1.1 mL·min^− 1^. Four flavonoids were detected at different wavelengths.

### Determination of volatile substances in tomato fruits

Using an electronic nose (PEN3 E-nose) (Airsense Analytics GmbH, Schwerin, Germany), equipped with 10 sensors, the signal response of the 10 sensors is represented as (G/G0), the ratio of the conductivity of volatile substances to the conductivity of pure air. Different sensors can detect different odor sensitive substances. The tomato fruit was ground into a homogenate, and 5 g was accurately weighed into a 20 mL brown headspace sample bottle. The anhydrous sodium sulfate (0.75 g) was sequentially added and stirred with a magnetic rotor, and the bottle cap was quickly tightened. Balance the gas in the bottle by heating it on a constant temperature magnetic stirrer (50℃) for 15 min, and finally insert a detection needle into the sample bottle to measure odor sensitive substances. The instrument detection parameters were as follows: sensor flushing 60s, sensor zeroing 10s, measurement time 180s. Each sample was tested three times in parallel. Analyze the sensor at the most stable signal time point [45]. The performance of the PEN3 electronic nose sensor array is shown in Table 1.

**Table 1:** Performance of PEN3 type electronic nose sensor array

Arram number	Sensor	Substances for sensing
R1	W1C	Aromatic compounds
R2	W5S	Nitrogen oxide
R3	W3C	Ammonia, aromatic molecules
R4	W6S	Hydride
R5	W5C	Short chain alkanes and aromatic components
R6	W1S	Alkanes
R7	W1W	Sulfide
R8	W2S	Alcohols, aldehydes, and ketones
R9	W2W	Aromatic components, organic sulfides
R10	W3S	Long chain alkanes

### Determination of relative gene expression levels

The extraction of RNA was carried out according to the instructions of the plant total RNA extraction kit (Tiangen Biochemical Technology Co., Ltd, Beijing, China). According to the instructions of the FastKing RT kit first strand synthesis kit (Tiangen, Beijing), reverse transcription was performed to synthesize complementary cDNA using 2 µL RNA as a template. Fluorescence quantitative analysis was performed using SYBR Green reagent kit (Tiangen) with a reaction system volume of 20 µL, including 2 µL cDNA solution, 10 µL 2 * SuperReal PreMix Plus, 10 µM forward and reverse primers 0.6 µL, 0.4 µL 50 * ROX Reference Dye Δ, and 6.4 µL distilled water. use LightCycler@480 Perform qRT PCR using the II real-time fluorescence quantitative PCR instrument. Calculate the relative expression levels of relevant genes using the 2^−∆∆Ct^ method. The relevant primer information is shown in Table 2.

**Table 2:** The primers information

Gene Name	Forward Primer Sequence (5’-3’)	Reverse Primer Sequence (5’-3’)
*HXK1*	GCACTATACAGAATACAGGATG	AATGAGAGGCAGCAAGAA
*HXK2*	ATCCAATACCTCCTTGAAGA	ATACAATCCGCCATCCAT
*FBP*	CTCTTGACACATCCTAACATC	ATTGCTACGCCACCATAT
*PK1*	CCTGCTGAGTCTACGAAT	ATAATCTTAACCACCGATGC
*PK2*	TGGTCAGGTGGAAGTTAT	CCAGAATCTCGGAAGGTA
*PEPCK*	TGGTCAGGTGGAAGTTAT	CCAGAATCTCGGAAGGTA
*SPS*	TATTCGTCCTTCCATTCTGA	GTCTTCATCCTCAACAACAA
*SS*	GCTCAAGGACAGGACTAA	GCTCATACATCTTCTTCATCTC
*AI*	CGGAATTGGATTGTGGAAT	CAGGTCAGCAGATTCACT
*NI*	GCGTATAATCACTGGTAGC	GAATCCACTGCCTTCTTAG
*PPC1*	TACAGCCTGAATGCCGAGTT	TCCTTGCCAGAGTCGGAGTA
*PPC2*	AGCCGTCCATCAAAGCGTAA	TTTTTGTGCATGTCCCGCAG
*cMDH*	GCCTGGTATGACCCGAGATG	GCAGCAATAGGGACAGTGGA
*CS*	CCAGGTATCTCAACACGGC	CGGCCAGCTCTTCAATAGGG
*Actin*	AGAACTATGAATTGCCTGATGGAC	TGAGCACAATGTTACCGTAGAGG

### Statistical analysis

Microsoft Excel 2020 was used for data organization and statistical analysis. SPSS 22.0 was used to perform One-Way ANOVA analysis with a significance level of *P* < 0.05 and multiple comparison using Duncan’s method. The results were presented in the format of “mean ± standard error”. Finally, Origin 2021 was used to create figures for the paper.

## Results

### Effect of different concentrations of EBR on the hardness of tomato fruits

**Fig. 1:**
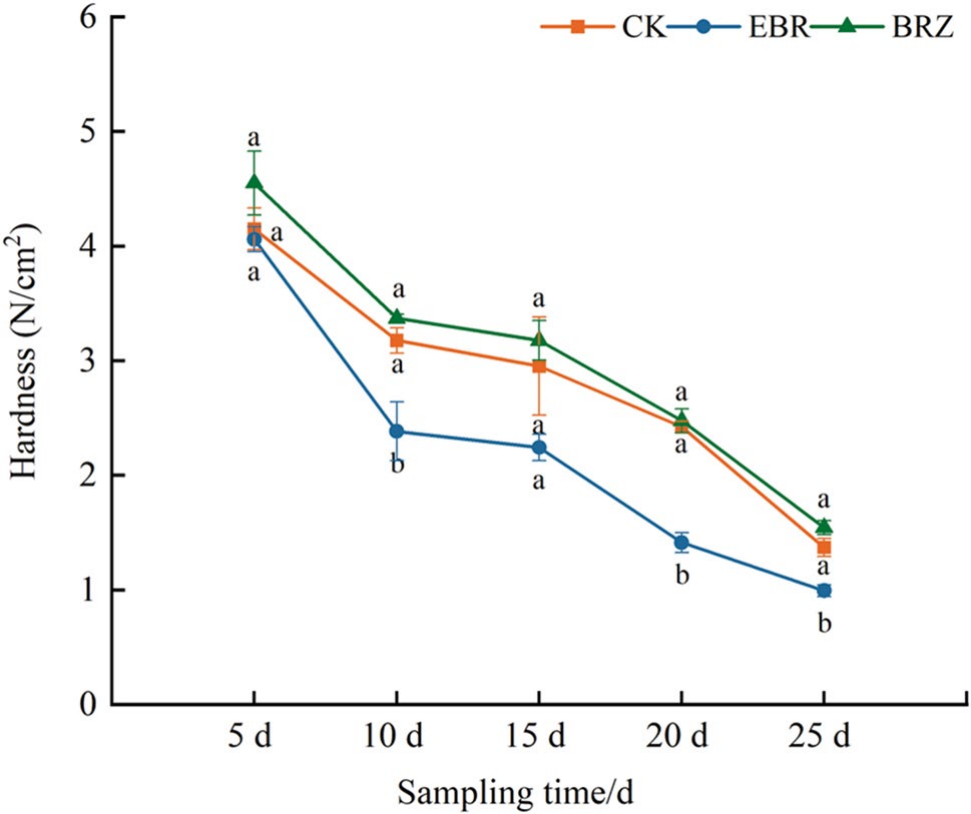
Variation of tomato fruit hardness. Fruits were treated at the green mature stage, with applications conducted every three days. CK (distilled water), EBR (0.15 mg/L EBR), and Brz (4 µmol/L Brz). Measurements of the specified parameters were taken on days 5, 10, 15, 20, and 25 following the treatment. Data indicates mean ± SE (*n* = 3). Significances were tested within the same day by one-way ANOVA. Different letters indicate significant differences (*P* < 0.05)

As shown in Fig. 1, as the fruit ripens, the fruit hardness gradually decreases, with EBR treatment showing the fastest decrease in fruit hardness and Brz treatment showing the slowest decrease. Compared with CK, EBR treatment significantly reduced fruit hardness by 25.16%, 41.74%, and 27.74% at 10, 20, and 25 d, while Brz treatment resulted in higher fruit hardness than CK at various time points, but there was no significant difference. The above results indicated that EBR treatment could accelerate fruit ripening and softening.

### Effect of different concentrations of EBR on soluble solids and vitamin C content of tomato fruits

**Fig. 2:**
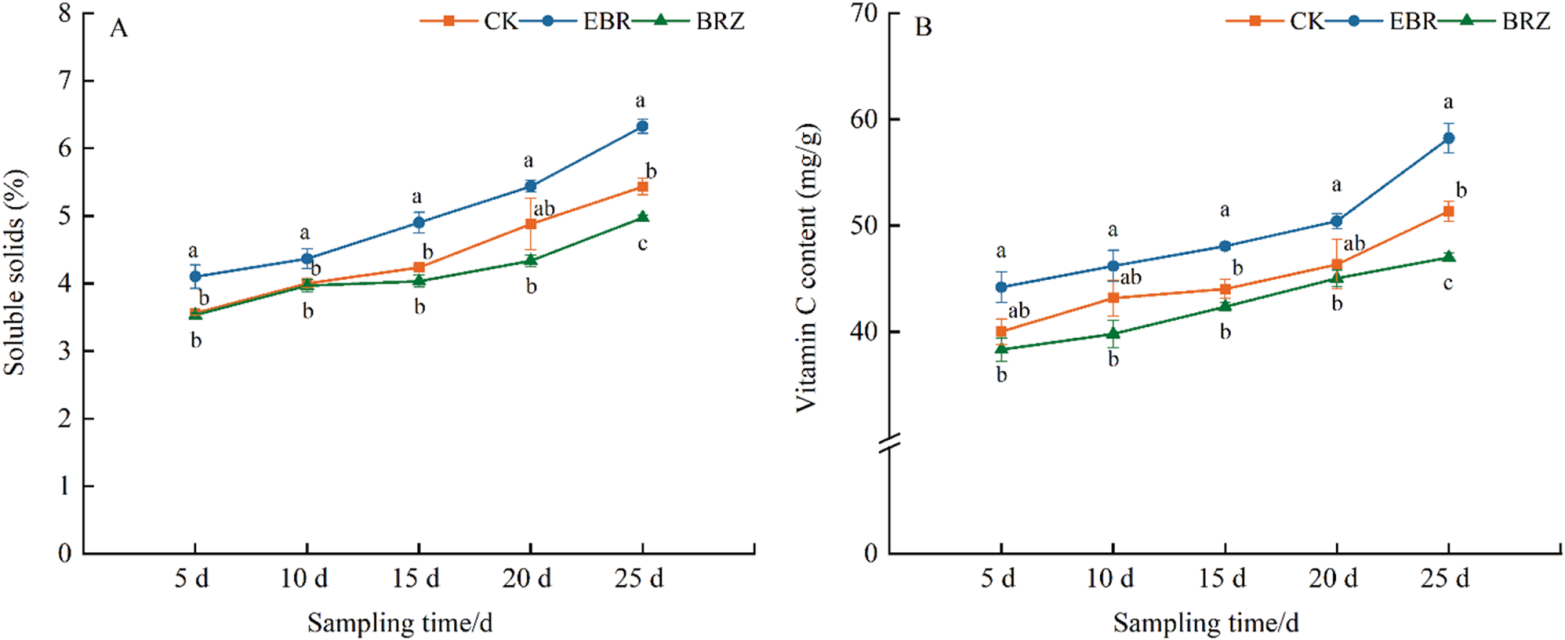
The effect of EBR treatment on (**A**) soluble solids and (**B**) VC content in tomato fruits. Fruits were treated at the green mature stage, with applications conducted every three days. CK (distilled water), EBR (0.15 mg/L EBR), and Brz (4 µmol/L Brz). Measurements of the specified parameters were taken on days 5, 10, 15, 20, and 25 following the treatment. Data indicates mean ± SE (*n* = 3). Significances were tested within the same day by one-way ANOVA. Different letters indicate significant differences (*P* < 0.05)

As shown in Fig. 2, with the extension of processing time, the content of soluble solids and VC in tomato fruits gradually increases, with EBR increasing the fastest, followed by CK, and Brz changing the least. Especially on the 25th day, the soluble solids and VC content of each treatment were the highest and there were significant differences between the treatments. Compared with CK and Brz treatments, EBR significantly increased the soluble solids content by 14.1% and 21.4%, and the VC content by 11.8% and 19.2%, respectively.

### Effect of exogenous application of different concentrations of EBR on the content of sugar components in tomato fruits

**Fig. 3:**
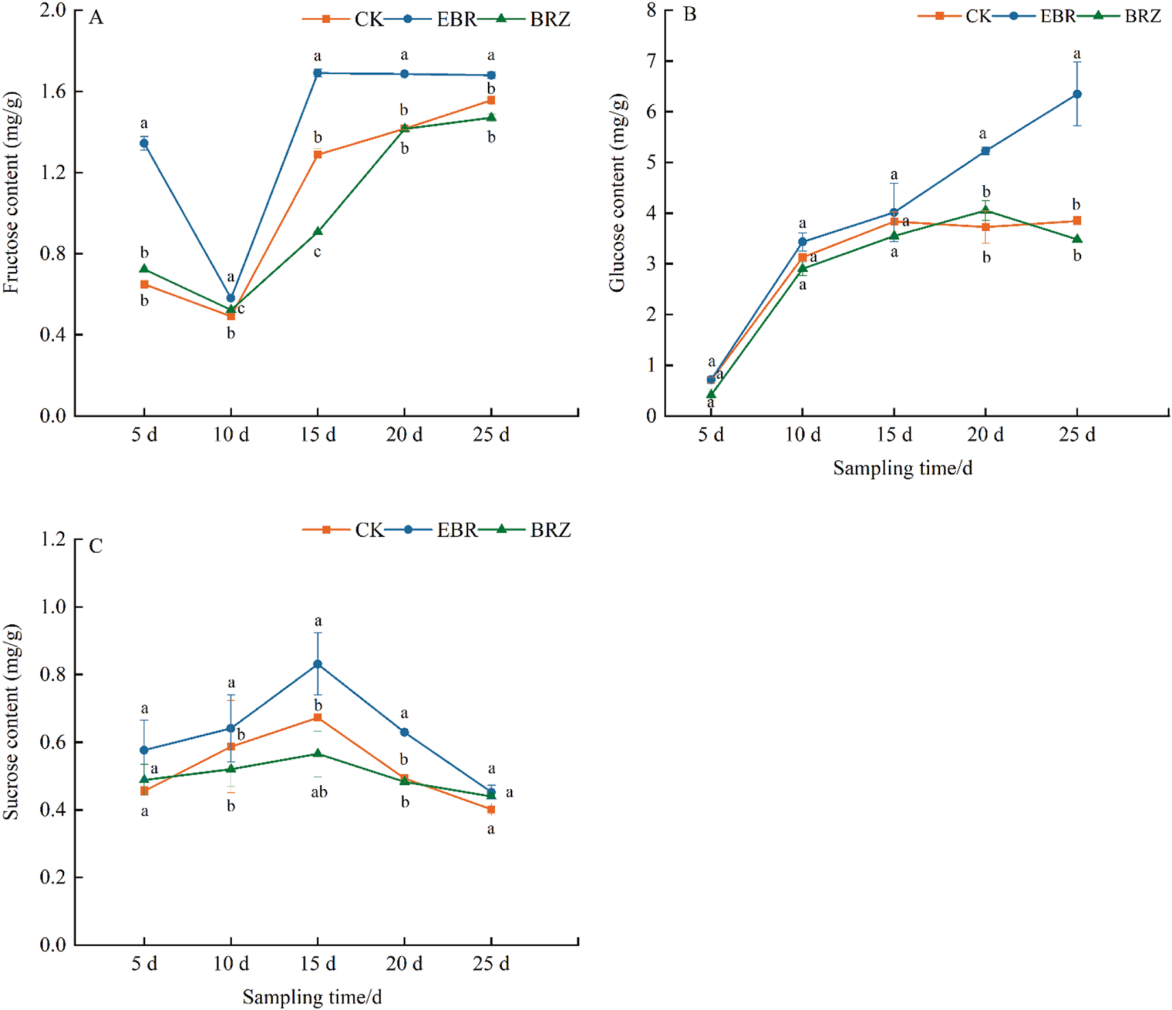
The effect of EBR treatment on the content of (**A**) fructose, (**B**) sucrose, and (**C**) glucose in tomato fruits. Fruits were treated at the green mature stage, with applications conducted every three days. CK (distilled water), EBR (0.15 mg/L EBR), and Brz (4 µmol/L Brz). Measurements of the specified parameters were taken on days 5, 10, 15, 20, and 25 following the treatment. Data indicates mean ± SE (*n* = 3). Significances were tested within the same day by one-way ANOVA. Different letters indicate significant differences (*P* < 0.05)

Three soluble sugars were detected in tomato fruits, including fructose, glucose, and sucrose. The content of fructose and glucose gradually increases with the ripening of tomato fruits (Fig. 3). EBR treatment could significantly promote the accumulation of fructose and glucose in tomatoes. At 25 days, the content of fructose and glucose reaches its highest value. Compared with CK treatment, EBR treatment significantly increases fructose and glucose by 23.67% and 39.53%. The sucrose content showed a trend of first increasing and then decreasing with the increase of treatment time (Fig. 3C). On the 15th day, the sucrose content in tomato fruit was the highest, and EBR significantly increased by 19.28% and 32.53% compared to CK and Brz. There was no significant difference between the treatments on the 25th day. These results indicated that EBR could increase fruit sugar content during tomato ripening, while Brz weaken this effect.

### The effect of exogenous EBR on the expression of sugar metabolism related genes in tomato fruits

**Fig. 4:**
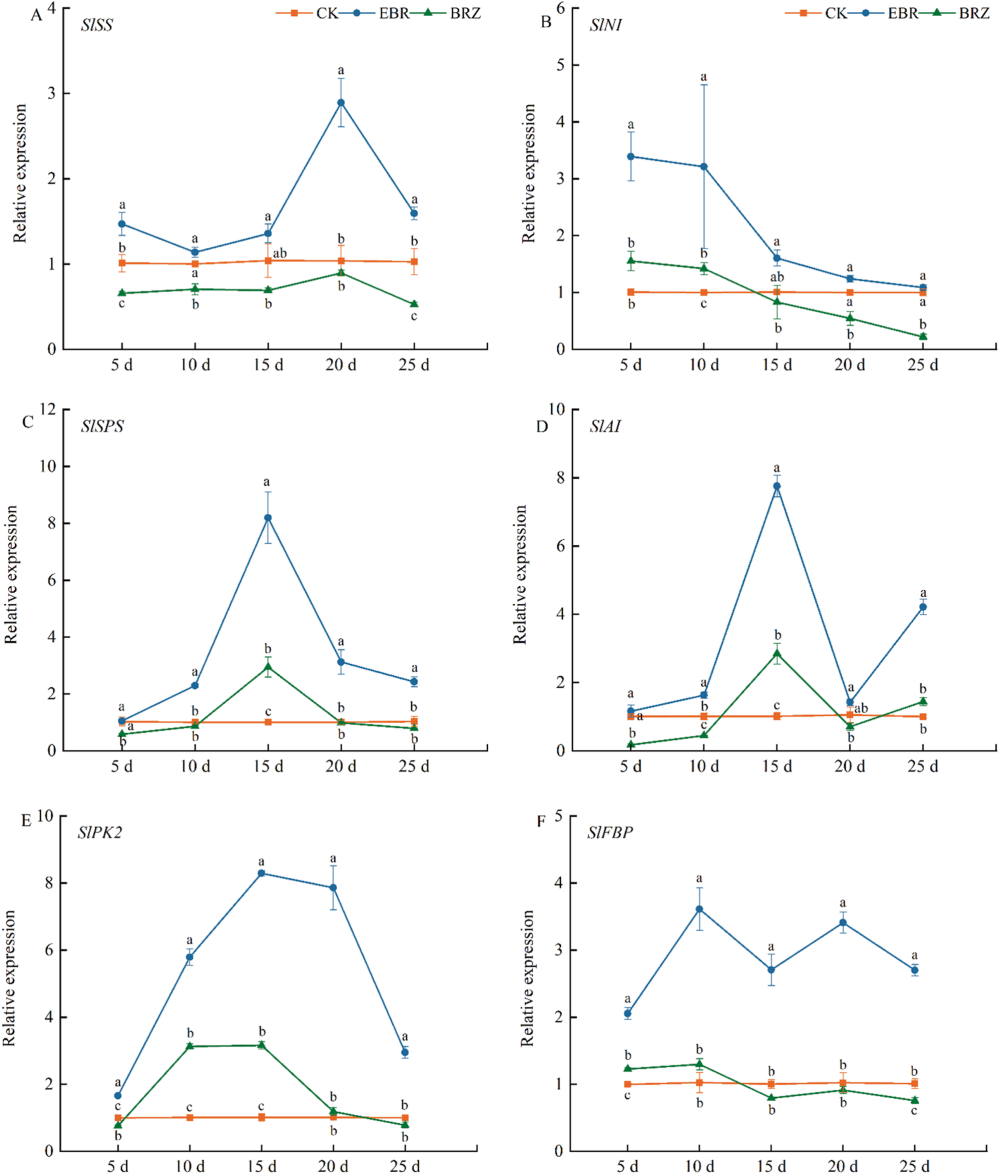

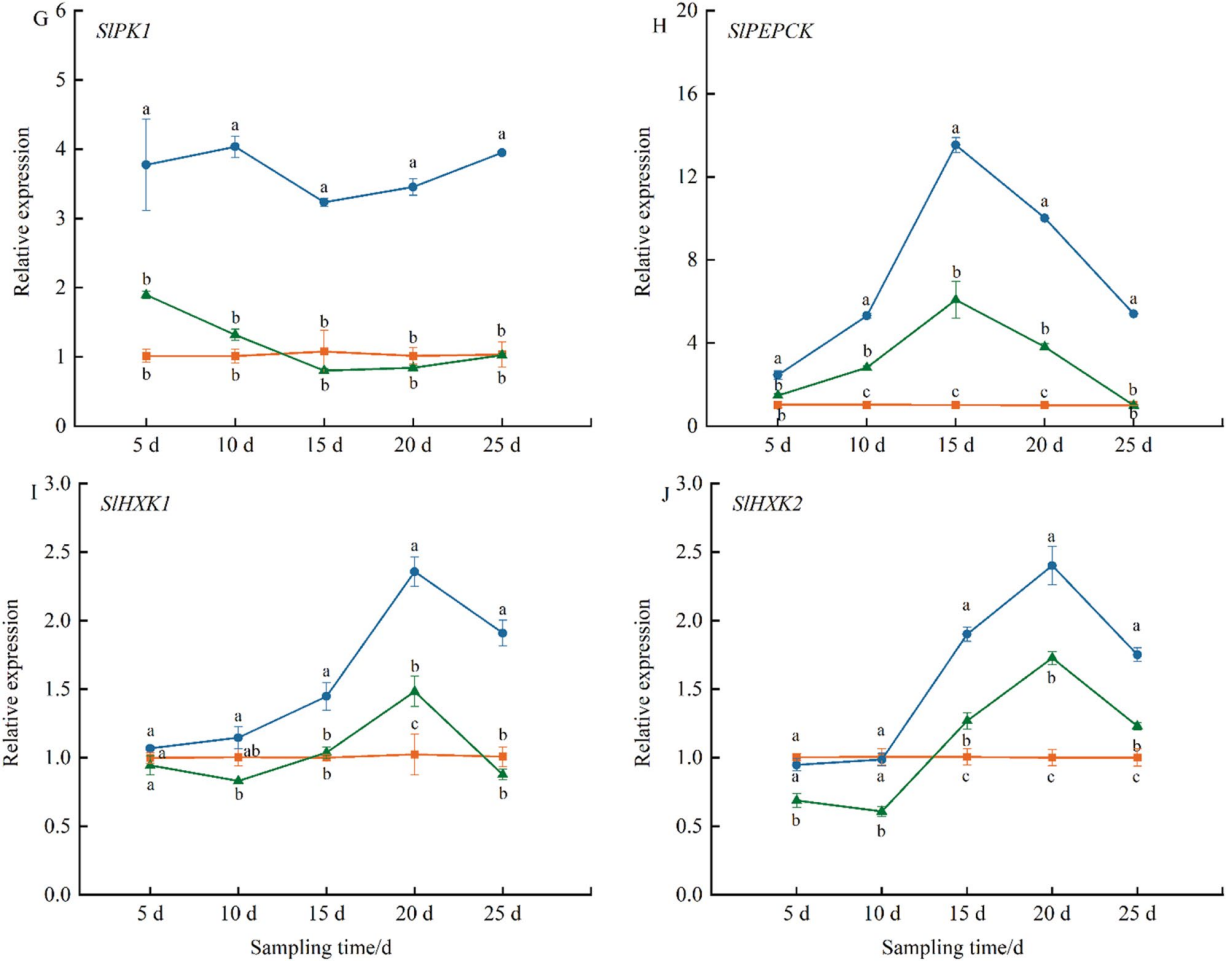
The effect of EBR treatment on gene expression of sugar metabolism pathway in tomato fruit. (**A**) *SlSS*, (**B**) *SlNI*, (**C**) *SlSPS*, (**D**) *SlAI*, (**E**) *SlPK1*, (**F**) *SlPK2*, (**G**) *SlPEPCK*, (**H**) *SlFBP*, (**I**) *SlHXK1* and (**J**) *SlHXK2* genes were determined. Fruits were treated at the green mature stage, with applications conducted every three days. CK (distilled water), EBR (0.15 mg/L EBR), and Brz (4 µmol/L Brz). Measurements of the specified parameters were taken on days 5, 10, 15, 20, and 25 following the treatment. Data indicates mean ± SE (*n* = 3). Significances were tested within the same day by one-way ANOVA. Different letters indicate significant differences (*P* < 0.05)

To investigate the effects of EBR on the expression of genes involved in sugar metabolism, we measured the expression levels of genes associated with sucrose, glucose, and fructose metabolism. As shown in Fig. 4A-D, *SS*, *NI*, *SPS*, and *AI* are key genes in sucrose metabolism. Under EBR treatment, the expression levels of *SS*, *NI*, *SPS*, and *AI* were higher at all time points compared to the control (CK), while Brz treatment resulted in significantly lower expression levels than EBR treatment. The expressions of *SS*, *NI*, *SPS*, and *AI* peaked at 20, 10, 15, and 15 d, respectively, indicating that sucrose was being accumulated and simultaneously broken down into fructose and glucose.

Figure 4E-H depicted the expression levels of glucose metabolism-related genes in tomato fruit. *SlPK2* and *PEPCK* showed a marked increase in expression between 10 and 20 d after EBR treatment, exhibiting 7.2-fold and 13.4-fold higher expression than CK, respectively. The expression of *PK1* and *FBP* was significantly higher than that of the control at all time points, while under Brz treatment, the expression levels of *SlPK1* and *FBP* were consistently lower than CK or showed no significant difference as the fruit matured.

Fig. 4I-J indicated that in the early stages of EBR treatment (5–10 days), there were no significant changes in the expression of *HXK1* and *HXK2*. However, during the fruit ripening phase, the expression of *HXK1* and *HXK2* began to rise significantly. Under Brz treatment, the expression levels of *HXK1* and *HXK2* were lower than CK, suggesting that Brz could partially counteract the effects of EBR

### Effect of exogenous application of different concentrations of EBR on the content of acid components in tomato fruits

**Fig. 5:**
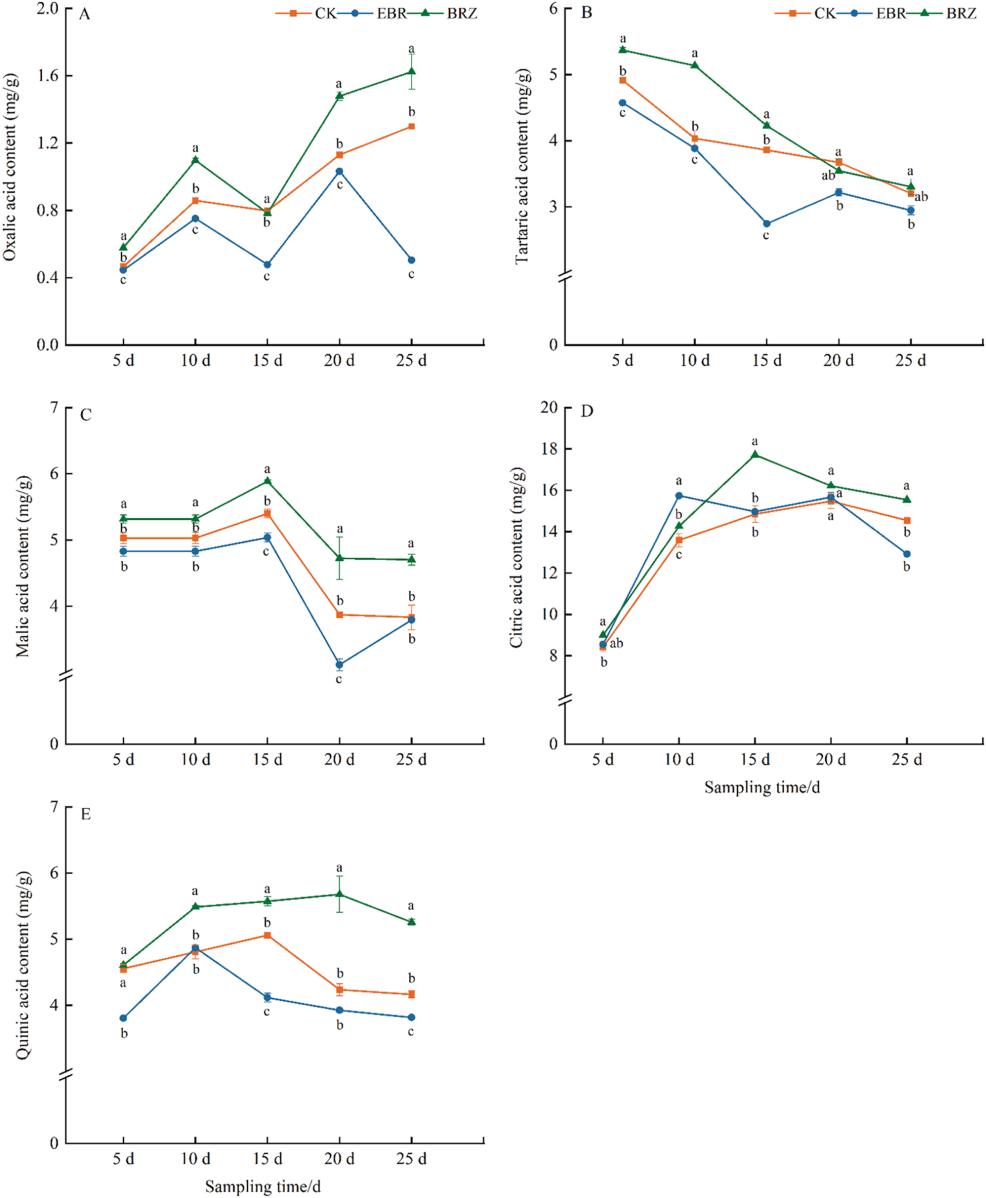
The effect of EBR treatment on the content of acid components in tomato fruits. (**A**) Oxalic acid, (**B**) tartaric acid, (**C**) malic acid, (**D**) citric acid, (**E**) quinic acid were determined. Fruits were treated at the green mature stage, with applications conducted every three days. CK (distilled water), EBR (0.15 mg/L EBR), and Brz (4 µmol/L Brz). Measurements of the specified parameters were taken on days 5, 10, 15, 20, and 25 following the treatment. Data indicates mean ± SE (*n* = 3). Significances were tested within the same day by one-way ANOVA. Different letters indicate significant differences (*P* < 0.05)

As shown in Fig. 5, the acid content in the fruit decreased progressively with maturation under EBR treatment, compared to CK, while Brz treatment resulted in higher acid content than both CK and EBR treatment. Malic acid and citric acid were the primary acids in tomato fruit. From 5 to 15 d after treatment, malic acid content remained relatively stable across all treatments, but fruits treated with EBR consistently exhibited lower malic acid levels than those under CK and Brz treatment. By day 20, the malic acid content in EBR-treated fruits was at its lowest, measuring 3.1 mg/g, significantly lower than both CK and Brz-treated fruits. Citric acid content initially increased as the fruit matured, followed by a decline, with the citric acid content in EBR-treated fruits being significantly lower than that of CK and Brz-treated fruits on day 25. These results indicated that EBR could reduce the content of acidic components in the fruit, while the use of Brz, by weakening endogenous BR function, promoted higher acid content.

### The effect of exogenous EBR on the expression of acid metabolism related genes in tomato fruits

**Fig. 6:**
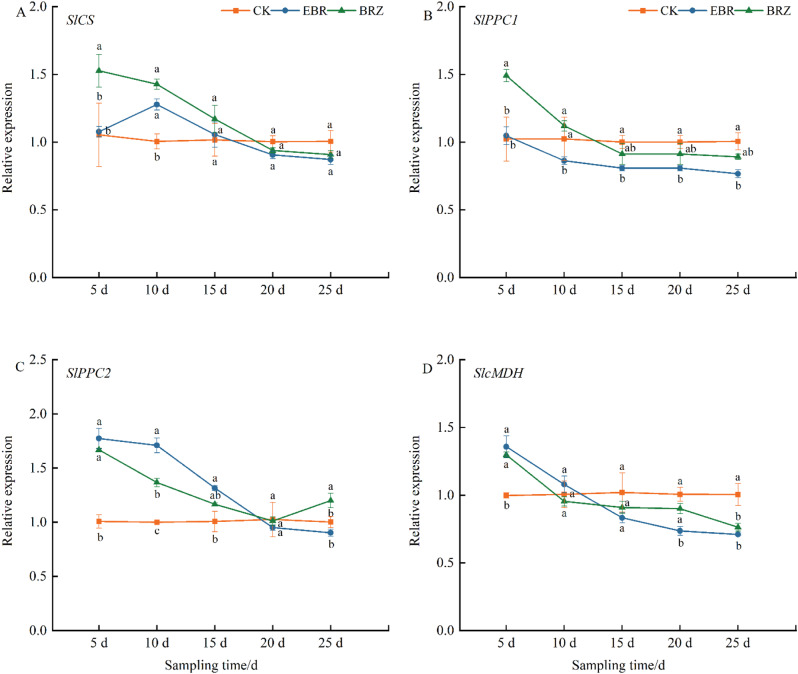
The effect of EBR treatment on gene expression of sugar metabolism pathway in tomato fruit. (**A**) *SlCS*, (**B**) *SlPC1*, (**C**) *SlPC2* and (**D**) *SlMDH* genes were determined. Fruits were treated at the green mature stage, with applications conducted every three days. CK (distilled water), EBR (0.15 mg/L EBR), and Brz (4 µmol/L Brz). Measurements of the specified parameters were taken on days 5, 10, 15, 20, and 25 following the treatment. Data indicates mean ± SE (*n* = 3). Significances were tested within the same day by one-way ANOVA. Different letters indicate significant differences (*P* < 0.05)

To investigate whether EBR reduces citric acid and malic acid content by inhibiting their metabolic pathways, we measured the expression changes of the *CS*, *PPC1*, *PPC2*, and *MDH* genes. The gene of *CS* (citrate synthase) promotes the synthesis of citric acid in the fruit, while *MDH* (malate dehydrogenase) and *PEPC* (phosphoenolpyruvate carboxylase) are key enzymes for malic acid synthesis. As shown in Fig. 6A, with the progression of treatment, the expression of *CS* gradually decreased under both EBR and Brz treatments compared to CK. The expression level of *CS* under Brz treatment was higher than CK and EBR from days 5 to 15, particularly during the early stages (5 d), where it was significantly elevated by 44.9% and 41.7%, respectively. Between days 20 and 25, no significant differences were observed between treatments. The expression of *PPC1* and *PPC2* also gradually decreased under both EBR and Brz treatments. On days 10 to 15, compared to CK, *PPC1* expression under EBR treatment decreased by 15.7%, 19.3%, 31.0%, and 23.9%, respectively. At days 5 and 10, Brz treatment led to a 45.8% and 9.5% increase compared to CK, and a 42.0% and 29.9% increase compared to EBR (Fig. 6B). The expression of *PPC2* under EBR treatment was significantly higher than CK at days 5, 10, and 15, showing increases of 76.1%, 71.0%, and 31.0%, respectively (Fig. 6C). At day 5, *MDH* gene expression was significantly upregulated under both EBR and Brz treatments compared to CK, but then steadily decreased over time, falling significantly below CK levels by day 25. These findings suggested that EBR reduces organic acid synthesis by downregulating the expression of genes associated with acid metabolism.

### The effect of exogenous EBR on the content of phenolic flavonoids in tomato fruits

To investigate the effect of exogenous EBR on the content of phenolic flavonoids in tomato fruits, the content of flavonoids and phenolic acid components in tomato fruits was measured on the 5, 10, 15, 20, and 25th day after treatment (Table 3). Four types of flavonoids were determined, namely rutin, quercetin, naringenin, and kaempferol. 12 phenolic acids, including protocatechuic acid, p-hydroxybenzoic acid, chlorogenic acid, gallic acid, 4-coumaric acid, ferulic acid, cinnamic acid, gentianic acid, caffeic acid, articarin acid, benzoic acid, and erucic acid.

**Table 3:** Comparison of EBR and Brz on flavonoid fractions and contents of tomato fruits

Flavonoid	Sampling time	CK	EBR	Brz
Rutin	5 d	317.57 ± 8.84a	334.31 ± 6.18a	233.80 ± 6.84b
	10 d	479.23 ± 2.55b	618.65 ± 6.64a	432.38 ± 14.35c
	15 d	403.07 ± 19.54b	555.89 ± 29.67a	425.84 ± 26.95b
	20 d	371.69 ± 17.41b	576.8 ± 18.55a	305.31 ± 18.65c
	25 d	528.76 ± 17.35b	711.23 ± 22.02a	259.56 ± 20.29c
Quercetin	5 d	204.53 ± 32.33a	291.00 ± 38.53a	200.12 ± 23.44a
	10 d	325.10 ± 6.65b	361.31 ± 9.89a	222.01 ± 6.53c
	15 d	247.64 ± 4.43b	337.53 ± 4.7a	245.44 ± 9.54b
	20 d	319.92 ± 7.18b	404.00 ± 5.85a	402.43 ± 8.25b
	25 d	354.65 ± 17.35b	435.08 ± 9.99a	332.45 ± 17.39b
Naringenin	5 d	9.20 ± 0.43a	9.19 ± 0.27a	7.28 ± 0.03b
	10 d	25.44 ± 0.88b	39.58 ± 0.87a	24.58 ± 0.58b
	15 d	71.85 ± 1.77ab	81.80 ± 5.65a	67.93 ± 1.36b
	20 d	114.26 ± 2.96a	128.38 ± 14.35a	111.02 ± 3.20a
	25 d	113.30 ± 4.75a	118.26 ± 4.32a	106.41 ± 1.27a
Kaempferol	5 d	1.66 ± 0.25a	2.27 ± 0.29a	0.74 ± 0.06b
	10 d	2.45 ± 0.22a	3.54 ± 0.41a	2.59 ± 0.27a
	15 d	4.56 ± 0.61ab	5.30 ± 0.37a	3.59 ± 0.14c
	20 d	5.32 ± 0.25a	5.62 ± 0.46a	4.56 ± 0.82a
	25 d	7.49 ± 0.40a	8.04 ± 0.61a	5.86 ± 0.19b

The results demonstrated that exogenous EBR significantly enhanced the accumulation of flavonoid compounds in tomato fruits, particularly rutin and quercetin, whose levels were consistently higher than those in the control and Brz-treated groups across all time points, peaking at 711.23 µg/g and 435.08 µg/g, respectively, on 25 d. EBR also markedly increased naringenin and kaempferol concentrations, especially on days 10 and 15 for naringenin. In contrast, Brz exhibited an inhibitory effect on the accumulation of these flavonoids, most notably in the reduced levels of rutin and quercetin compared to both the control and EBR-treated groups, suggesting an antagonistic regulatory role to EBR in flavonoid metabolism. Overall, EBR treatment substantially enhanced flavonoid content in tomato fruits, indicating its potential to improve their nutritional value.

### The effect of exogenous EBR and Brz on the volatile compounds in tomato fruits

**Fig. 7:**
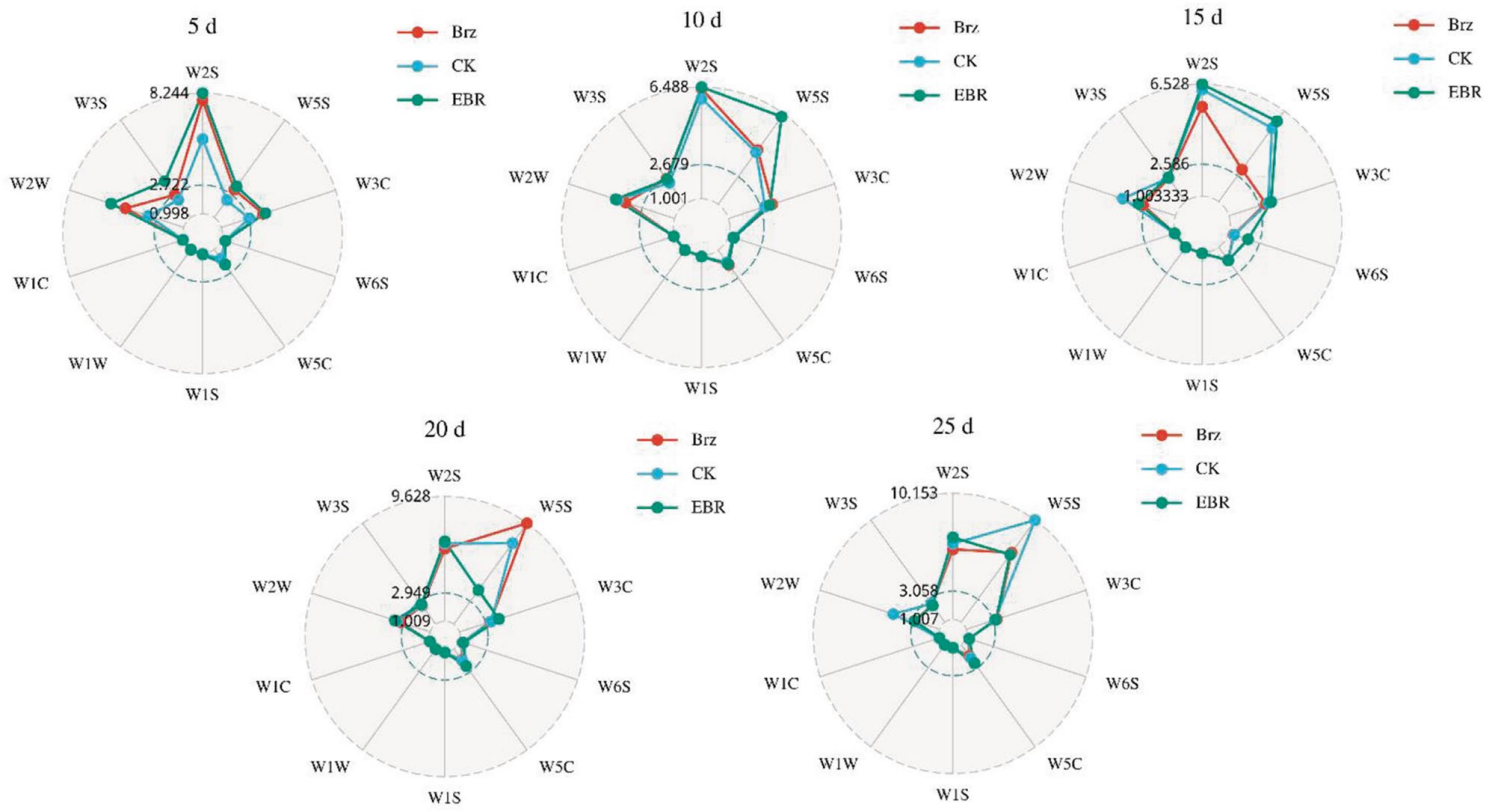
The effect of exogenous EBR on the contribution value of odor characteristics

As shown in Fig. 7, the radar chart revealed the differences in the contribution values of 10 sensors in the electronic nose to volatile compounds in tomato fruit under CK, EBR, and Brz treatments. On day 5, the sensors W1C (aromatic compounds), W2W (aromatic components, organic sulfides), W3C (ammonia, aromatic molecules), and W3S (long chain alkanes) displayed higher response values compared to other sensors. Additionally, most sensors showed a significantly increased response under EBR and Brz treatments compared to CK. By day 10, sensors W2W and W5S exhibited higher response values under EBR treatment compared to CK and Brz, with W2W, W5S, W1C, and W3C showing response values above 3, which were higher than those of other sensors. On day 15, W2W and W5S had the highest response values among all sensors, with the W5S sensor in the Brz treatment group showing significantly higher response levels than the other treatments. On day 20, sensors W5S and W2W still exhibited high response values, with the W5S sensor under CK treatment showing a notably higher response level. By day 25, W2W, W5S, W1C, and W3C maintained high response values, with the response values under EBR treatment being significantly higher than those of CK and Brz. These results indicated that W2W (nitrogen oxides) and W5S (aromatic components, organic sulfides) could significantly respond to EBR induction in the middle and late stages of fruit ripening, and also suggested that EBR may have a positive impact on the synthesis of nitrogen oxides, aromatic components, and organic sulfides.

### Comprehensive analysis of the impact of EBR on tomato fruit quality

In order to comprehensively evaluate the improvement effect of EBR on tomato quality, we conducted principal component analysis and heatmap analysis. When conducting principal component analysis, two principal components were obtained. Principal component 1 mainly included hardness soluble solids, vitamin C, fructose, glucose, oxalic acid, tartaric acid, citric acid, quinic acid, rutin, quercetin, naringenin and kaempferol. Principal component 2 included sucrose and malic acid. According to Table 4, the ranking of comprehensive scores is EBR > CK > Brz.

**Table 4:** Comprehensive rating ranking of the impact of different treatments on tomato quality

Treatment	Principal Component 1		Principal Component 2	Score	Ranking
CK		0.52578	-1.02805	0.322849802	2
EBR		0.62743	0.96937	0.672087364	1
Brz		-1.15321	0.05868	-0.994937166	3

**Fig. 8:**
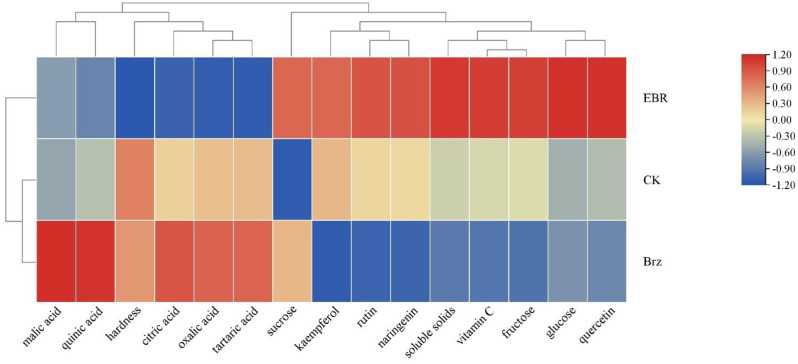
Cluster heatmap of tomato quality indicators

The heat map analysis showed the influence of different treatments on tomato quality indicators (Fig. 8). EBR treatment has a significant effect on key qualities such as vitamin C, sugar components, and flavonoids in tomatoes, while Brz has the opposite effect. Overall, EBR treatment was more effective in promoting the content of important quality indicators in tomatoes than CK and Brz treatments.

## Discussion

Fruit quality is a critical indicator of economic value, and its enhancement represents a pivotal aspect of agricultural production [46, 47]. BR have been extensively studied for their role in enhancing crop yield and quality [48]. Tomato firmness is a key quality trait closely associated with storage durability; however, excessive firmness can compromise flavor and texture quality [49]. Tomato fruit ripening is predominantly regulated by ethylene. Recent studies have demonstrated that brassinosteroids (BRs) promoted ethylene biosynthesis and carotenoid accumulation through direct targeting of ethylene and carotenoid biosynthetic genes by BZR1, thereby ensuring proper fruit ripening and quality formation [33, 50]. In this study, we observed that tomato fruit firmness was significantly lower than the control at various time points under EBR treatment, while Brz treatment resulted in higher firmness compared to the control (Fig. 1). Additionally, the contents of soluble solids and vitamin C gradually accumulated during tomato maturation, with higher levels observed under EBR treatment across all stages compared to the control (Fig. 2). These findings indicated that EBR promoted tomato fruit ripening and softening while enhancing nutritional quality, potentially through the interaction between BR and ethylene Hu et al. demonstrated that overexpression of *SlCYP90B3* increased BR content in tomatoes, resulting in enhanced fruit softening, elevated soluble sugar levels, and improved flavor compound accumulation [51]. In grapes, exogenous BR application had also been shown to reduce fruit firmness [52, 53]. Studies have shown that the application of BR could improve the flavor of citrus fruits by increasing the levels of soluble solids, vitamin C, and total sugars [54]. Similarly, in tomatoes, the application of high levels of BR could enhance fruit yield, soluble solids content, and ascorbic acid (vitamin C) levels [42]. Similar results have also been observed in strawberries [55].

The sugar components in tomato fruits primarily include fructose, sucrose, and glucose, while the acid components mainly consist of malic acid, citric acid, oxalic acid, succinic acid, and tartaric acid.

 [56]. Studies have shown that exogenous application of BR (3.0 µmol L^− 1^) induces changes in the sugar metabolism of mung bean sprouts, increasing the levels of glucose, fructose, sucrose, and phosphoenolpyruvate. Furthermore, enhanced enzyme activities associated with sucrose metabolism and glycolysis were detected in the BR-treated group [57]. During grape fruit maturation, EBR treatment increased the levels of soluble sugars, glucose, and fructose, while reducing organic acid content. Combined treatment with EBR and GA₃ enhances the accumulation of alcohols, esters, ketones, terpenes, and phenolics in tomato aroma compounds, as well as the levels of glucose and fructose, a phenomenon similarly observed in apples [31, 58]. Xu et al. also found that EBR significantly increased the glucose and fructose content in *Vitis vinifera* ‘Cabernet Sauvignon’ berries by 55.40% and 78.08%, respectively, while Brz treatment led to a decrease in glucose and sucrose content by 3% [59]. EBR treatment in combination with jasmonic acid (JA) also promoted glucose accumulation and enhances antioxidant capacity [60]. Under alkaline stress, the content of malic acid and citric acid in the roots of *Malus hupehensis* significantly increased, but decreased after exogenous BR application [61]. Foliar application of 1 µM and 3 µM 24-epibrassinolide reduced the total titratable acid in strawberry fruit flesh, increased the soluble solids content, and improved the sugar/acid ratio [55]. Similar results were observed in this study. Our experiment showed that, during the 15–25 d period, EBR treatment significantly increased the contents of sucrose, fructose, and glucose in tomato fruits, especially fructose and glucose (Fig. 3). In contrast, the content of acid components was progressively reduced under EBR treatment, with a significant decrease in oxalic acid, citric acid, and malic acid in the later stages of fruit maturation (Fig. 5). Under Brz treatment, sugar accumulation in tomato fruits was inhibited, while acid component levels were higher than in the EBR treatment. These results suggested that EBR promoted sugar accumulation in tomato fruits, reduced acid content, and improved the sugar-to-acid ratio, whereas Brz inhibited this transformation.

There are also relevant reports on the regulation of sugar and acid metabolism by BR. Treatment with 0.4 mg L^− 1^ EBL in grapes significantly increased the activity of acid invertase (INV) and neutral invertase (NI), while Brz notably reduced the activity of both enzymes [62]. Furthermore, compared to the control group, treatment with 0.6 mg L^− 1^ exogenous EBL significantly enhanced the activity of sucrose-phosphate synthase (SPS) during grape maturation, while markedly upregulating the activity of cell wall acid invertase (VvcwINV), sucrose transporter (VvSUC12), and sucrose synthase (VvSS) [63]. EBR treatment also elevated the transcriptional levels of genes encoding enzymes involved in sucrose metabolism (*VvcwINV*), monosaccharide transporters (*VvHT3*, *4*, *5*, and *6*), and disaccharide transporters (*VvSUC12* and *27*), resulting in an increase in soluble sugar content [59]. Our study, by examining the transcription levels of key genes in both sugar and acid metabolic pathways, demonstrated that EBR significantly reduced the synthesis of citric and malic acids, while promoting the accumulation of sucrose and its conversion into glucose and fructose, thereby enhancing the sugar metabolism process (Figs. 4 and 6). Although Brz treatment initially upregulated the expression of some genes associated with acid and sugar metabolism, it later inhibited their expression, partially counteracting the regulatory effects of EBR. These findings indicated that EBR optimizes fruit quality by downregulating genes involved in acid metabolism and upregulating those related to sugar metabolism, thereby balancing fruit acidity and sugar content.

Flavonoids are important secondary metabolites in plants, known for their diverse physiological functions. They play a significant role in the prevention and treatment of cancer, cardiovascular diseases, diabetes, inflammation, and aging [64]. Flavonoids in tomato fruit primarily include rutin, naringenin, and quercetin [65, 66]. Most studies show that flavonoids are important antioxidants in plants. In maize, treatments with HBL and EBL increased the total flavonoid content by 31.56% and 31.09%, respectively, enhanced free radical scavenging activity by 37.99% and 77.41%, and reduced oxidative damage caused by salt stress [67]. Salt stress reduced the flavonoid content in tomatoes by 52.64%. However, supplementation with EBL, Ca, and EBL + Ca under NaCl stress further increased the flavonoid content by 15.31%, 8.12%, and 56.66%, respectively [68]. Moreover, crosstalk between BR signaling and ROS signaling has been shown to influence the levels of flavonoids such as kaempferol and quercetin in tomato plants, thereby enhancing their resistance to stress [69]. Exogenous BR increased the levels of flavonoids and nitric oxide (NO) in a concentration-dependent manner, whereas the BR biosynthesis inhibitor Brz reduced the concentrations of flavonoids and NO in tea leaves [70]. Comparable findings have been reported in research conducted on strawberries [71]. In this study, exogenous EBR significantly enhanced the accumulation of flavonoids in tomato fruits, particularly rutin and quercetin, which peaked at 711.23 µg/g and 435.08 µg/g at 25 d, respectively. Additionally, EBR markedly increased the concentrations of naringenin and kaempferol. In contrast, Brz exhibited an inhibitory effect on flavonoid accumulation, with rutin and quercetin levels significantly lower than those in the control and EBR-treated groups (Table 3). These findings indicated that EBR promoted flavonoid accumulation, playing a crucial role in enhancing plant resilience against stress.

**Fig. 9:**
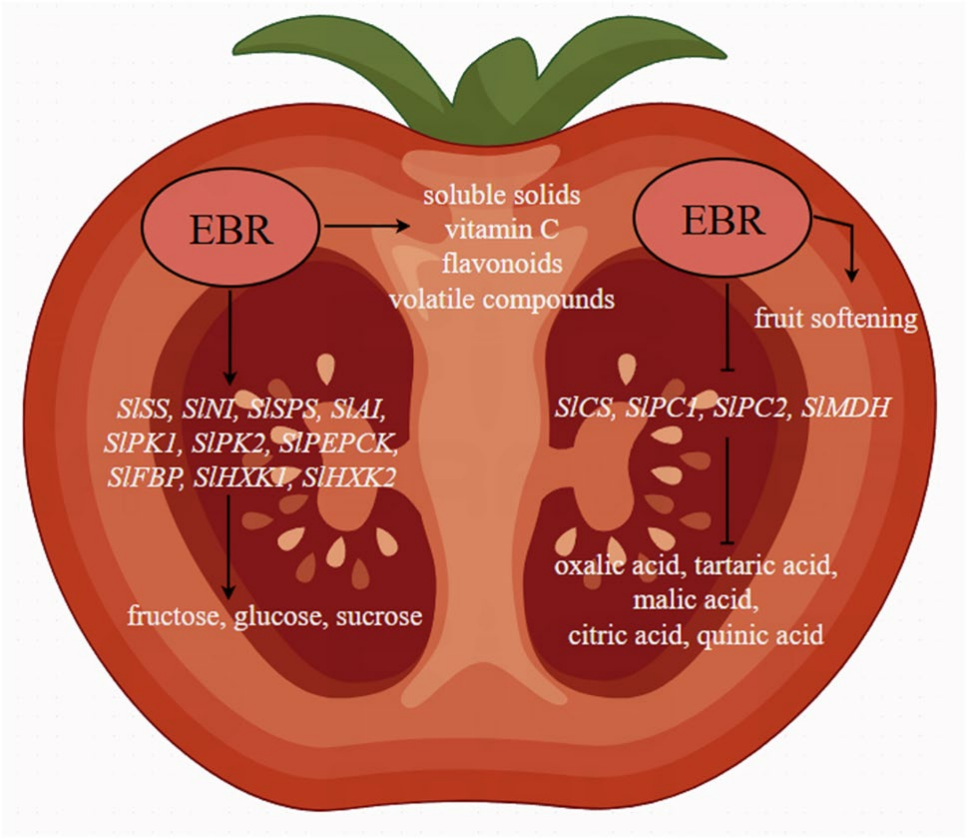
Schematic diagram of EBR regulation of tomato quality. Hexokinase (HXK); Sucrose synthase (SS); Alkaline/neutral invertase (NI); Sucrose phosphate synthase (SPS); Acid invertase (AI); Pyruvate kinase (PK); Fructose-1,6-bisphosphate (FBP); Phosphoenolpyruvate carboxykinase (PEPCK); Citrate synthase (CS); Phosphoenolpyruvate carboxylase (PC); Malate dehydrogenase (MDH)

Numerous studies have demonstrated that BRs influence the content and composition of plant volatile compounds. Treatment with 24-epibrassinolide (EBL) significantly altered the volatile profile of tomato fruits by modulating key gene expression levels. Specifically, EBL enhanced the accumulation of 6-methyl-5-hepten-2-one (MHO) through the upregulation of *SlPSY1* and *CCD1A*. Furthermore, it increased the concentration of salicylaldehyde while reducing methyl salicylate (MeSA) levels by upregulating *SALD* and *SAMT* while downregulating *SABP2*. Weighted gene co-expression network analysis (WGCNA) of 2473 differentially expressed genes and 27 volatiles identified 11 volatile-associated modules, along with hub genes within these networks [72]. Exogenous BR promoted the accumulation of various aroma compounds in grapes by upregulating the expression of key genes involved in the biosynthesis of terpenes, aldehydes, and alcohols [73]. Additionally, ABA + BR treatment modulated volatile composition in grape, promoting the accumulation of alcohols (geraniol) and aldehydes (trans-2-hexenal), increasing the diversity of ketones and alkanes, and enhancing ester content (ethyl acetate), thereby improving aroma quality [74]. BRs could influenc flavonoid metabolism, unsaturated fatty acid biosynthesis, and terpen metabolic pathways under cold storage conditions. ABA and BR synergistically regulated this process by enhancing the expression of genes such as *FAD*, *LOX*, *TPS*, and *HMGR*, thereby preserving the flavor quality of grapes during storage [75]. Similarly, radar chart analysis revealed significant differences in the sensor responses to volatile compounds in tomatoes under CK, EBR, and Brz treatments. Sensors W2W and W5S consistently showed high responsiveness, with EBR treatment significantly enhancing sensor responses, particularly during the later stages of fruit development. These findings highlight the substantial impact of EBR on the volatile compound profile of tomatoes (Fig. 7). Overall, EBR could improve the nutritional and flavor quality of tomato fruits, and its mechanism of action is shown in Fig. 9.

## Conclusions

In summary, exogenous application of EBR accelerated tomato fruit ripening and enhanced fruit quality by modulating sugar-acid metabolism and promoting the accumulation of flavonoids and volatile aroma compounds. EBR upregulated key genes in sugar metabolism (e.g., *SS*, *NI*) while suppressing acid metabolism genes (e.g., *CS*, *MDH*), resulting in increased sweetness and reduced acidity. Additionally, EBR enhanced nutritional value by boosting flavonoid content and improved sensory attributes through altered volatile profiles. Conversely, BR biosynthesis inhibition (Brz treatment) exerted opposite effects, underscoring the critical role of BR signaling in fruit quality regulation. Further studies are required to elucidate the molecular mechanisms by which EBR regulates tomato fruit quality through signal transduction and to explore the potential of BR in enhancing crop yield and quality.

## Data Availability

No datasets were generated or analysed during the current study.
